# Controllability in Hybrid Kinetic Equations Modeling Nonequilibrium Multicellular Systems

**DOI:** 10.1155/2013/274719

**Published:** 2013-09-26

**Authors:** Carlo Bianca

**Affiliations:** Dipartimento di Scienze Matematiche, Politecnico, Corso Duca degli Abruzzi 24, 10129 Torino, Italy

## Abstract

This paper is concerned with the derivation of hybrid kinetic partial integrodifferential equations that can be proposed for the mathematical modeling of multicellular systems subjected to external force fields and characterized by nonconservative interactions. In order to prevent an uncontrolled time evolution of the moments of the solution, a control operator is introduced which is based on the Gaussian thermostat. Specifically, the analysis shows that the moments are solution of a Riccati-type differential equation.

## 1. Introduction

The derivation of mathematical models for the evolution of multicellular systems is a challenge of the last and also this century. Several mathematical frameworks have been proposed with the aim of capturing the main properties of these systems, which have also the feature to be complex [1]. Indeed different emerging behaviors appear as result of the interactions: competition, pattern formation, and organization (see the book [2] and the review paper [3]).

The mathematical frameworks present in the pertinent literature principally pertain to multicellular (biological) systems that are not subjected to external forces (systems at equilibrium); the interested reader is referred to the recent review [4] (and the references cited therein) for what concern the hyperbolic and kinetic models for living systems at equilibrium.

However, dealing with multicellular systems whose time evolution depends on the presence of external fields requires a different treatment. An external force field moves the system away from equilibrium and the blow-up of some observable (such as the kinetic energy) occurs.

Recently a new mathematical framework has been proposed and analyzed: the thermostated kinetic theory for active particles [5–9]. This framework, which refers to general complex systems (including vehicular traffic, and crowd and swarm dynamics; see, among others, [10–13]), is based on kinetic theory for active particles and deterministic thermostat. Kinetic equations coupled with the Gaussian isokinetic thermostat are already present in the literature: Kac equation [14–16] and Boltzmann equation with the one-dimensional BGK operator [17] (see also the recent review paper [18]). The solution of these equations is a distribution (probability) function defined in the microscopic state of the particles, which can include mechanical variables (space and velocity) and/or biological variables.

Roughly speaking, the deterministic thermostat is a mathematical framework that has been proposed in nonequilibrium molecular dynamics in order to achieve equilibration to nonequilibrium situation where there is a flux of energy through the system induced by external fields. The reader interested in further details is referred to papers [19, 20] and the review paper [21]. Accordingly, a control term (a damping term) is inserted into the equations of motion in order to ensure the conservation of the kinetic energy (or a different moment); see [22, 23].

The thermostated kinetic for active particles framework proposed in [7] is concerned with nonequilibrium complex systems characterized by conservative interactions only (namely, interactions that only modify the microscopic state of the particles). Therefore, the mathematical modeling of nonequilibrium multicellular systems, where proliferative and destructive events may occur, cannot directly performed within this framework.

This paper is concerned with a further generalization of the thermostated kinetic framework [7] that takes into account the modeling of multicellular systems with nonconservative interactions. Moreover, the velocity variable is discretized, generating an hybrid framework with discrete and continuous variables. Therefore, the new thermostated kinetic framework consists of nonlinear hybrid partial integrodifferential equations with quadratic nonlinearity.

It is worth stressing that, to the best of our knowledge, this is the first time that nonconservative interactions are taken into account in the thermostated kinetic for active particles framework.

Specifically, in the present paper the time evolution of the moments is analyzed. The control term, which is based on the mathematical thermostats, allows the derivation of nonlinear ordinary differential equations (Riccati-type equations) fulfilled by the moments.

The framework proposed here is certainly worth of future research activity concerning both its qualitative analysis and the application to modelling complex biological systems; see [7] and the references cited therein. Mathematical control in integrodifferential equations is already presented in the pertinent literatur; see, among others, [24].

The contents of the present paper are divided into three sections which follow this introduction.  Section 2 highlights the essential hybrid mathematical settings.  Section 3 deals with the analysis of the moments evolution. Finally, Section 4 is meant to future research perspectives.

## 2. The Underlying Hybrid Kinetic Framework

This section is meant to a concise description of an hybrid kinetic for active particle framework that constitutes a new paradigm for the modelling of complex multicellular systems characterized by nonconservative particle interactions. Specifically, these systems are composed by cells (active particles), with different genotypes and phenotypes, which are able to perform a task; for example, the immune system is constituted by cells that protect the human body from pathogens, and the connective tissue is made up of fibroblast cells that synthesize the extracellular matrix and collagen.

The mathematical structure consists of autonomous partial integrodifferential equations system with quadratic nonlinearity constituted by three different operators: the operator which models conservative interactions, the operator that takes care of nonconservative interactions, and the operator that ensures the control of the evolution of the moments. Each equation is a kinetic-type equation whose solution is the distribution function *f*(*t*, **x**, **v**, *u*) of cells that at time *t* ∈ [0, *∞*[, possess the triplet of microscopic variables (**x**, **v**, *u*), where **x** ∈ *D*_**x**_ ⊂ ℝ^3^ is the space variable, **v** ∈ *D*_**v**_ ⊂ ℝ^3^ is the velocity variable, and *u* ∈ *D*_*u*_ ⊂ ℝ is the task-variable (activity). The elementary product *f*(*t*, **x**, **v**, *u*) *d* **x** *d* **v** *du* is the number of cells whose state, at time *t*, is in the elementary volume of the space of microscopic states *dΩ* = [**x**, **x** + *d* **x**]×[**v**, **v** + *d* **v**]×[*u*, *u* + *du*]. Let *Ω* = *D*_**x**_ × *D*_**v**_ × *D*_*u*_ be the domain of the all possible microscopic states and

(1) $$\begin{array}{c} d\mu_{\Omega }=dx\;dv\;du \end{array}$$

the Lebesgue measure on *Ω*. If *f* ∈ *L* ^1^[*Ω*, *dμ*_*Ω*_], then the following expectation *𝔼*_0_[*f*] of *f*:

(2) $$\begin{array}{c} {𝔼}_{0}\left[f\right]\left(t\right)=\int_{\Omega }f\left(t,x,v,u\right)d\mu_{\Omega } \end{array}$$

represents the global number, at time *t*, of the cells. 

Higher order moments of *f* can be defined under suitable integrability assumption on *f*. Let *ω*_*m*,*n*,*p*_ = |**x**|^*m*^ | **v**|^*n*^ | *u*|^*p*^, where |·| denotes the Euclidean distance of · and *m*, *n*, *p* ∈ *ℕ*. If, for instance, *ω*_*m*,*n*,*p*_ *f* ∈ *L* ^1^[*Ω*, *dμ*_*Ω*_], the (*m*, *n*, *p*)th-order moment of *f* is defined as follows:

(3) $$\begin{array}{c} {𝔼}_{m,n,p}\left[f\right]\left(t\right)=\int_{\Omega }\omega_{m,n,p}f\left(t,x,v,u\right)d\mu_{\Omega }. \end{array}$$

It is worth stressing that if *f* represents the joint distribution function, marginal distribution functions of *f* refer either to the distribution over the mechanical state or to distribution over the microscopic activity. These marginal distribution functions define the local quantities. For instance, the local quadratic activity moment at time *t* in **x** is computed as follows:

(4) $$\begin{array}{c} {𝔸}_{2}\left[f\right]\left(t,x\right)=\int_{D_{v}\times D_{u}}u^{2}f\left(t,x,v,u\right)dv\;du \end{array}$$

and represent the energy expressed by the activity variable.

The task dynamics performed by cells is modified, at the time *t*, by *binary interactions*, which occur at the microscopic level and refer to the mutual actions between the cell with microscopic state (**x**, **v**, *u*) (test cell) and the cell with microscopic state (x*, v*, u*) (field cell), or the test cell and the cell with microscopic state (**x**_∗_, **v**_∗_, *u*_∗_) (candidate cell). The candidate cell is the cell that reaches the microscopic state of the test cell after the interaction with the field cell.

### 2.1. The Hybrid Kinetic Setting at Equilibrium

Looking at the motion of cells, it is nonrestrictive to assume that velocity variables can attain discrete values **v** ∈ {**v**_1_, **v**_2_,…, **v**_*n*_} and the activity variable is continuous *u* ∈ *D*_*u*_. Therefore, the cell microscopic state includes both discrete and continuous variables. Moreover, we assume space homogeneity. Accordingly, the distribution function *f* of the system reads

(5) $$\begin{array}{c} f\left(t,v,u\right)=\overset{n}{\underset{i=1}{\sum }}f\left(t,v_{i},u\right)\delta \left(v-v_{i}\right)=\overset{n}{\underset{i=1}{\sum }}f_{i}\left(t,u\right)\delta \left(v-v_{i}\right). \end{array}$$

The mathematical framework thus consists of a set of evolution equations for *f*_*i*_(*t*, *u*), which represents the distribution function of the test cell. Let *f*_*i*_(*t*, *u*_∗_) be the distribution function of the candidate cell and *f*_*i*_(*t*, u*) the distribution function of the field cell.

The time evolution of the distribution function of test cell *f*_*i*_(*t*, *u*) is modified when the candidate cell, with task-variable *u*_∗_, after the interaction with the field cell, with task-variable *u*_∗_, can acquire the task-variable *u*. The interactions in multicellular systems are *conservative* (modify the magnitude activity variable of the cells) and *nonconservative* (that modify the number of cells as a consequence of proliferation and destruction events occurring for natural birth/death and competition). Since *f*_*i*_(*t*, *u*)*du* denotes the number of cells, at time *t*, such that *u* ∈ [*u*, *u* + *du*], then

(6) $$\begin{array}{c} f_{i}\left(t,u_{*}\right)du_{*}f_{j}\left(t,u^{*}\right)du^{*} \end{array}$$

is a nonlinear product that refers to the interaction, at time *t*, between the number of candidate cells, with velocity **v**_*i*_ such that *u*_∗_ ∈ [*u*_∗_, *u*_∗_ + *du*_∗_] and the number of field cells, with velocity **v**_*j*_, such that u* ∈ [u*, u* + du*]; the possibility of this interaction can be measured by introducing the nonnegative function *ι*_*ij*_(*u*_∗_, u*) which represents the interaction rate; the probability that after this interaction the candidate cell undergoes a change in its microscopic state (that of test particle) is measured by the nonnegative function *𝒜*_*ij*_(*u*_∗_, u*, *u*), which is a probability density with respect to *u* and then

(7) $$\begin{array}{c} \int_{D_{u}}{𝒜}_{ij}\left(u_{*},u^{*},u\right)du=1,\;\forall u_{*},u^{*}\in D_{u}. \end{array}$$

Bearing all above in mind, the (infinitesimal) result of the interaction reads

(8) $$\begin{array}{c} {𝒜}_{ij}\left(u_{*},u^{*},u\right)\iota_{ij}\left(u_{*},u^{*}\right)f_{i}\left(t,u_{*}\right)du_{*}f_{j}\left(t,u^{*}\right)du^{*}, \end{array}$$

and summing up with respect to all the candidate and field cells, we obtain the following operator which models the gain of test cell:

(9) 𝒢ij[fi,fj](t,u)  =∫Du×Duιij(u∗,u∗)𝒜ij(u∗,u∗,u)      ×fi(t,u∗)fj(t,u∗)du∗du∗.

Similarly, the loss of test cells is modeled by the following operator:

(10) $$\begin{array}{c} {ℒ}_{ij}\left[f_{i},f_{j}\right]\left(t,u\right)=f_{i}\left(t,u\right)\int_{D_{u}}\iota_{ij}\left(u,u^{*}\right)f_{j}\left(t,u^{*}\right)du^{*}. \end{array}$$

Finally, if *π*_*ij*_(*u*, u*) is the net birth/death rate of the test cell due to the interaction with the field cell, then the operator which models nonconservative interactions reads

(11) 𝒩ij[fi,fj](t,u)=fi(t,u)∫Duιij(u,u∗)      ×πij(u,u∗)fj(t,u∗)du∗.

The evolution equation of the distribution function *f*_*i*_ over the microscopic state can be derived by a balance equation of the inlet and outlet flows in the elementary volume [*u*, *u* + *du*] of the space of the microscopic states. The hybrid kinetic for active particles framework thus reads:

(12) ∂tfi(t,u)=∑j=1n(𝒢ij[fi,fj](t,u)   −ℒij[fi,fj](t,u)+𝒩ij[fi,fj](t,u)).

**Definition 1**

Let *ι*_*ij*_(*u*_1_, *u*_2_) : *D*_*u*_ × *D*_*u*_ → ℝ^+^, for *i*, *j* ∈ {1,2,…, *n*}, be the interaction rate between the *u*_1_-cell distributed according to *f*_*i*_(*t*, *u*_1_) and the *u*_2_-cell distributed according to *f*_2_(*t*, *u*_2_). Let *𝒜*_*ij*_(*u*_1_, *u*_2_, *u*) : *D*_*u*_ × *D*_*u*_ × *D*_*u*_ → ℝ^+^ be the probability density satisfying the property (7). A function *f*_*i*_ = *f*_*i*_(*t*, *u*):(0, *∞*) × *D*_*u*_ → ℝ^+^ is said to be the solution of (12) if

- *f*_*i*_(*t*, *u*) ∈ *C*((0, *∞*), *L* ^1^(*D*_*u*_));
- *f*_*i*_ is differentiable with respect to the variable *t*;
- *ι*_*ij*_(*u*_1_, *u*_2_)*𝒜*_*ij*_(*u*_1_, *u*_2_, *u*)*f*_*i*_(*t*, *u*_1_)*f*_*j*_(*t*, *u*_2_) is an integrable function with respect to the elementary measure *du*_1_ *du*_2_;
- *ι*_*ij*_(*u*_1_, *u*_2_)*f*_*j*_(*t*, *u*_2_) is an integrable function with respect to the elementary measure *du*_2_;
- *ι*_*ij*_(*u*_1_, *u*_2_)*π*_*ij*_(*u*_1_, *u*_2_)*f*_*j*_(*t*, *u*_2_) is an integrable function with respect to the elementary measure *du*_2_;
- *f*_*i*_ satisfies (12) for all (*t*, *u*)∈(0, *∞*) × *D*_*u*_.

Setting **f** = (*f*_1_(*t*, *u*), *f*_2_(*t*, *u*),…, *f*_*n*_(*t*, *u*)) ∈ ℝ^*n*^, the (*p*, *q*)-order moment of the distribution function *f*(*t*, **v**, *u*), for *p*, *q* ∈ *ℕ*, is written as follows:

(13) $$\begin{array}{c} {𝔼}_{p,q}\left[f\right]\left(t\right)=\overset{n}{\underset{i=1}{\sum }}v_{i}^{p}\int_{D_{u}}u^{q}f_{i}\left(t,u\right)du. \end{array}$$

In particular, the zero-order *𝔼*_0,0_ (density or mass), first-order *𝔼*_1,1_ (mean activation or linear momentum), and second-order *𝔼*_2,2_ (activation energy or kinetic energy) moments fulfill an important role depending on the system under consideration.

### 2.2. The Controlled Hybrid Kinetic Setting at Nonequilibrium

 The mathematical framework (12) is concerned with multicellular systems at equilibrium. Nonequilibrium conditions occur when the system is subjected to external fields *F*_*i*_(*u*) : *D*_*u*_ → ℝ^+^ at macroscopic scale. In this case, the kinetic framework reads

(14) ∂tfi(t,u)+∂u(Fi(u)fi(t,u)) =∑j=1n(𝒢ij[fi,fj](t,u)−ℒij[fi,fj](t,u)    +𝒩ij[fi,fj](t,u)).

The external field does work on the system thereby moving it away from equilibrium. Therefore, it follows uncontrolled increases of the activation energy (kinetic energy). The action of the Gaussian isokinetic thermostat is modeled, according to the Gauss' principle of least constrain [20, 25], by the following operator:

(15) $$\begin{array}{c} {𝒯}_{i}\left[F_{i},f\right]\left(t,u\right)=uF_{i}\left(u\right)f_{i}\left(t,u\right)\overset{n}{\underset{i=1}{\sum }}v_{i}\int_{D_{u}}uf_{i}\left(t,u\right)du \end{array}$$

which is a damping operator (thermostat operator) that is adjusted so as to control the activation energy. The introduction of the thermostat operator modifies the mathematical framework as follows:

(16) ∂tfi(t,u)+∂u(Fi(u)fi(t,u)−𝒯i[Fi,f](t,u)) =∑j=1n(𝒞ij[fi,fj](t,u)+𝒩ij[fi,fj](t,u)),

where *𝒞*_*ij*_[*f*_*i*_, *f*_*j*_](*t*, *u*) = *𝒢*_*ij*_[*f*_*i*_, *f*_*j*_](*t*, *u*) − *ℒ*_*ij*_[*f*_*i*_, *f*_*j*_](*t*, *u*) is the operator for the conservative interactions. In what follows, we refer to framework (16) as the controlled kinetic framework with conservative and nonconservative interactions.

**Definition 2**

Let *F*_*i*_ = *F*_*i*_(*u*), *u* ∈ *D*_*u*_, be an external force field differentiable with respect to *u*; *ι*_*ij*_(*u*_1_, *u*_2_) : *D*_*u*_ × *D*_*u*_ → ℝ^+^, for *i*, *j* ∈ {1,2,…, *n*}, interaction rate between the *u*_1_-cell distributed according to *f*_*i*_(*t*, *u*_1_) and the *u*_2_-cell distributed according to *f*_2_(*t*, *u*_2_); consider *𝒜*_*ij*_(*u*_1_, *u*_2_, *u*) : *D*_*u*_ × *D*_*u*_ × *D*_*u*_ → ℝ^+^ to be the probability density satisfying the property (7). A function *f*_*i*_ = *f*_*i*_(*t*, *u*):(0, *∞*) × *D*_*u*_ → ℝ^+^ is said to be the solution of the model (16) if

- *f*_*i*_(*t*, *u*) ∈ *C*((0, *∞*), *L* ^1^(*D*_*u*_));
- *f*_*i*_ is differentiable with respect to the variables *t* and *u*;
- *uf*_*i*_ is an integrable function with respect to the elementary measure *du*;
- *ι*_*ij*_(*u*_1_, *u*_2_)*𝒜*_*ij*_(*u*_1_, *u*_2_, *u*)*f*_*i*_(*t*, *u*_1_)*f*_*j*_(*t*, *u*_2_) is an integrable function with respect to the elementary measure *du*_1_ *du*_2_;
- *ι*_*ij*_(*u*_1_, *u*_2_)*f*_*j*_(*t*, *u*_2_) is an integrable function with respect to the elementary measure *du*_2_;
- *ι*_*ij*_(*u*_1_, *u*_2_)*π*_*ij*_(*u*_1_, *u*_2_)*f*_*j*_(*t*, *u*_2_) is an integrable function with respect to the elementary measure *du*_2_;
- *𝒯*_*i*_[*F*_*i*_, **f**] is differentiable with respect to the variable *u*;
- *f*_*i*_ satisfies (16) for all (*t*, *u*)∈(0, *∞*) × *D*_*u*_.

**Remark 3**

The theorem of existence and uniqueness of the solution for the controlled kinetic framework (16) has been obtained in [7] when the nonconservative operator *𝒩*_*ij*_ is equal to zero (conservative interactions only). The proof of the theorem can be adapted in order to obtain existence and uniqueness of the solution also for the nonconservative interactions case. Nevertheless, global existence may not occur. This is a work in progress and results will be reported in due course.

The depicted hybrid controlled kinetic framework (16) is quite general and can be exploited to originate specific models for multicellular systems by acting on the specific forms of the grid velocity, interaction rate *ι*_*ij*_, the probability density *𝒜*_*ij*_, the net rate of birth/death *π*_*ij*_, and the external force *F*_*i*_.

## 3. Differential Equations for the Moments

This section is concerned with the derivation of differential equations for the moments. Let *𝔼*_1_[**f**] be the following moment:

(17) $$\begin{array}{c} {𝔼}_{1}\left[f\right]\left(t\right)≔{𝔼}_{1,1}\left[f\right]\left(t\right)=\overset{n}{\underset{i=1}{\sum }}v_{i}\int_{D_{u}}uf_{i}\left(t,u\right)du. \end{array}$$

Let *μ*(*t*) be the following function:

(18) $$\begin{array}{c} \mu \left(t\right)≔\overset{n}{\underset{i=1}{\sum }}\mu_{i}\left(t\right), \end{array}$$

where

(19) $$\begin{array}{c} \mu_{i}\left(t\right)≔\int_{D_{u}}f_{i}\left(t,u\right)du, \end{array}$$

(20) $$\begin{array}{c} \bar{\mu }\left(t\right)≔\overset{n}{\underset{i=1}{\sum }}v_{i}\mu_{i}\left(t\right). \end{array}$$

The following result holds true.

**Theorem 4**

Let *ι*_*ij*_ = *ι*, *π*_*ij*_ = *π*, and *F*_*i*_ = *F* be real constants. If there exists a nonnegative solution **f** of the controlled kinetic framework (16) such that *f*_*i*_(*t*, *u*) = 0 as *u* ∈ ∂*D*_*u*_, then the 1th-order moment *𝔼*_1_[**f**](*t*) is solution of the following Riccati nonlinear ordinary differential equation:

(21) ddt𝔼1[f](t)=F(μ¯(t)−(𝔼1[f](t))2) −ι(1−π)μ(t)𝔼1[f](t).

**Proof**

The interaction operator *𝒥*[*f*_*i*_, *f*_*j*_] can be written as follows:

(22) 𝒥ij[fi,fj](t,u)=𝒢ij[fi,fj](t,u) −ιμj(t)fi(t,u)+ιπμj(t)fi(t,u).

Multiplying both sides of *𝒥*_*ij*_[*f*_*i*_, *f*_*j*_] by *u* and integrating over *D*_*u*_, we have

(23) ∫Duu𝒥ij[fi,fj](t,u)du   =−ιμj(t)(1−π)∫Duufi(t,u)du.

Summing with respect to *j*, multiplying by **v**_*i*_, and summing with respect to *i*, we obtain

(24) ∑i=1nvi∑j=1n∫Duu𝒥ij[fi,fj](t,u)du  =−ιμ(t)(1−π)𝔼1[f](t).

Multiplying by *u* and **v**_*i*_ the second term of the left hand side of (16), integrating with respect to the activity variable, performing integration by parts and summing with respect to *i*, we have

(25) ∑i=1nvi∫Duu∂u((1−u𝔼1[f](t))fi(t,u))du  =(𝔼1[f](t))2−μ¯(t)

and then the proof.

According to Theorem 4, the solution of the Riccati equation (21) can be obtained as follows. The Riccati equation reads

(26) ddt𝔼1[f](t)+F(𝔼1[f](t))2  +ι(1−π)μ(t)𝔼1[f](t)−Fμ¯(t)=0.

Setting

(27) $$\begin{array}{c} \gamma_{1}\left(t\right)=F,\;\;\gamma_{2}\left(t\right)=\iota \left(1-\pi \right)\mu \left(t\right),\;\;\gamma_{3}\left(t\right)=-F\;\bar{\mu }\left(t\right), \end{array}$$

if $\bar{{𝔼}_{1}[f](t)}$ is a solution of (26), the general integral can be written as

(28) $$\begin{array}{c} {𝔼}_{1}\left[f\right]\left(t\right)=\bar{{𝔼}_{1}\left[f\right]\left(t\right)}+\frac{1}{\lambda \left(t\right)}, \end{array}$$

where *λ*(*t*) is solution of

(29) $$\begin{array}{c} \lambda^{\prime }-\left[\gamma_{2}\left(t\right)+2\gamma_{1}\left(t\right)\bar{{𝔼}_{1}\left[f\right]\left(t\right)}\right]\lambda =\gamma_{1}\left(t\right). \end{array}$$

A nonnegative and constant solution of (26) is

(30) $$\begin{array}{c} \bar{{𝔼}_{1}[f](t)}=\frac{\sqrt{\iota^{2}{\left(1-\pi \right)}^{2}\mu^{2}\left(t\right)+4F^{2}\bar{\mu }\left(t\right)}-\iota \left(1-\pi \right)\mu \left(t\right)}{2F}. \end{array}$$

Therefore, the solution of (26) can be written as follows:

(31) $$\begin{array}{c} {𝔼}_{1}\left[f\right]\left(t\right)=\bar{{𝔼}_{1}\left[f\right]\left(t\right)}+\frac{e^{\int_{0}^{t}\beta (\tau )d\tau }}{{\left({𝔼}_{1}\left[f\right]\left(0\right)-\bar{{𝔼}_{1}\left[f\right]\left(t\right)}\right)}^{-1}+F\int_{0}^{t}e^{\beta (\tau )d\tau }}, \end{array}$$

where

(32) $$\begin{array}{c} \beta \left(\tau \right)=\sqrt{\iota^{2}{\left(1-\pi \right)}^{2}\mu^{2}\left(\tau \right)+4F^{2}\bar{\mu }\left(\tau \right)}. \end{array}$$

The next theorem gives the evolution equation for all moments where *p* is an odd number.

**Theorem 5**

Let *p* ∈ *ℕ*, *q* be an odd number and *t* ≥ 0. Then, the (*p*, *q*)th-order moment of the distribution function *f* satisfies the following Riccati nonlinear ordinary differential equation:

(33) ddt𝔼p,q[f](t)=pF𝔼p,q−1[f](t)(μ~(t)−𝔼p,q[f](t)) +ιμ(t)(1−π)𝔼p,q[f](t),

where

(34) $$\begin{array}{c} \tilde{\mu }\left(t\right)=\overset{n}{\underset{i=1}{\sum }}v_{i}^{p}\mu_{i}\left(t\right). \end{array}$$

Moreover, if   *𝔼*_*p*,*q*_[**f**](*t*) is initially bounded, it remains bounded for all *t* > 0.

**Proof**

The proof follows by multiplying both sides of (14) by *u* ^*p*^ and performing integration by parts on the control term.

## 4. Research Perspectives

 The controlled kinetic framework proposed in this paper allows the derivation of specific models for multicellular systems characterized by nonconservative interactions. This framework belongs to the class of thermostated kinetic for active particles models.

The mathematical framework (16) can be further generalized in order to include the role of mutations; see Nowak [26]. This is an important issue in the cancer modeling [27, 28].

 A future research perspective is the generalization of the mathematical framework (16) to open systems subjected to external actions at the microscopic scale, for example, the role that the outer environment has in the whole dynamics [29].

 Perspectives include also the introduction of stochastic terms that model jump processes in the activity or in the velocity variable; see paper [30] and the references section.

 Moreover, the proof of the existence of solutions to the stationary problem

(35) ∂u(Fi(u)fi(u)−𝒯i[Fi,f](u))   =∑j=1n(𝒞ij[fi,fj](u)+𝒩ij[fi,fj](u))

is missing. This proof has been gained for the conservative interactions case; see [31].

 An important research perspective is the development of suitable asymptotic limits for deriving macroscopic equations for the evolution of moments; see [17] and the references section. The derivation of these equations is based on suitable assumptions on the operators in order to obtain convergence results. Optimization investigation can also be performed on the solutions of these macroscopic equations, such as regularization and embedding results [32].

 It is worth stressing that the analysis of the moments performed in this paper can be straightforwardly applied also for the thermostated framework proposed in [9].
